# Effect of Bridge Position Swallow on Esophageal Motility in Healthy Individuals Using High-Resolution Manometry

**DOI:** 10.1007/s00455-020-10169-0

**Published:** 2020-08-04

**Authors:** Kei Aoyama, Kenjiro Kunieda, Takashi Shigematsu, Tomohisa Ohno, Ichiro Fujishima

**Affiliations:** 1 The Department of Rehabilitation Medicine, Chikamori Rehabilitation Hospital, 2-22 Nijudaimachi, Kochi, 780-0843 Kochi Japan; 2 Department of Neurology, Gifu University Graduate School of Medicine, Gifu, Gifu Japan; 3 The Department of Rehabilitation Medicine, Hamamatsu City Rehabilitation Hospital, Hamamatsu, Shizuoka Japan; 4 The Department of Rehabilitation Medicine, Seirei Awaji Hospital, Awaji, Hyogo Japan; 5 The Department of Dentistry, Hamamatsu City Rehabilitation Hospital, Hamamatsu, Shizuoka Japan

**Keywords:** High-resolution manometry, Gravity, Bridge position, Esophageal motility, Deglutition, Deglutition disorders

## Abstract

Recently, there has been clinical interest in the effect of different body positions on esophageal motility. This study aimed to identify the effect of three different body positions on esophageal motility using high-resolution manometry. Thirteen healthy adults swallowed 5 mL of water in the upright, supine, and bridge positions. For the bridge position, each subject raised their waist against gravity, placed a cushion under their back, and bent their knees. The proximal contractile integral (PCI) and distal contractile integral (DCI), integrated relaxation pressure (IRP), distal latency (DL), peristaltic breaks (PBs), intrabolus pressure (IBP), and expiratory and inspiratory esophagoesophageal junction (EGJ) pressure were measured. In the bridge position, PCI, DCI, IRP, and expiratory and inspiratory EGJ pressure were significantly higher than those in the upright position (bridge PCI vs. upright PCI [*p* = 0.001], bridge DCI vs. upright DCI [*p* < 0.001], bridge IRP vs. upright IRP [*p* = 0.018], bridge EGJ pressure vs. upright EGJ pressure [expiratory: *p* = 0.001] [inspiratory: *p* < 0.001]). PBs were significantly shorter and DL was significantly longer in the bridge position compared to upright (bridge PBs vs. upright PBs [*p* = 0.001], bridge DL vs. upright DL [*p* = 0.001]). IBP was significantly higher in the bridge position compared to supine (bridge IBP vs. supine IBP [*p* = 0.01]). These results demonstrated changes in esophageal motility according to changes in position while swallowing, where esophageal contractions became stronger against gravity. Further study is required to examine the effectiveness of swallowing in the bridge position.

## Introduction

Swallowing involves four phases: oral preparatory, oral propulsive, pharyngeal, and esophageal [1]. In dysphagia therapy, there are many treatments for the oral and pharyngeal phases [2, 3]; however, swallowing rehabilitation treatments for the esophageal phase have not been established. We focused on the effect of body position and gravity on esophageal motility in order to enhance esophageal contractility. Previous studies have reported that esophageal peristaltic pressure in the supine position is significantly higher than in the upright position [4–9]. It has also been pointed out that the effect is similar to patients with esophageal dysphagia and gastroesophageal reflux disease (GERD) [10], where gravity has been demonstrated to assist in distal esophageal bolus movement in the upright position [11].

High-resolution manometry (HRM) is a relatively new method for assessing the characteristics of esophageal pressure [12] and can be used to measure pressure from the pharynx to the stomach via closely spaced pressure transducers. Moreover, the use of solid-state pressure sensors instead of the conventional water-perfused pressure sensors enables a faster response to changes in pressure. This results in a more detailed assessment and makes it possible to study the relationships between pressure in the stomach, lower esophageal sphincter, and esophageal body in detail [13].

Although the normal eating position is upright, the accepted protocol and classification of esophageal motor disorders are indicated only for the supine position [14, 15]. Using HRM, a previous study investigated positional adaptations against gravity by evaluating swallowing pressure patterns in participants while in a series of inverted body positions [16]. However, the study was unable to comment on pressure changes in the distal esophagus due to the limited catheter length used. De Leon et al. [17] identified that the lower esophageal sphincter (LES) pressure significantly increased in the Trendelenburg position, which is an adjusted supine position with the patient’s head tilted lower than their feet; however, they did not investigate the distal esophageal peristalsis. Zifan et al. [18] investigated the effects of swallowing between the supine and Trendelenburg positions on the movement of the bolus, distension, and contractions of the esophagus in normal healthy subjects. They identified that esophageal contractile integrals, including those of the distal esophagus, were significantly higher in the Trendelenburg position than in the supine. In the study, a stretcher or inversion table was necessary for positions against gravity, while in the present study, a novel approach using the bridge position was explored. In addition, this study investigates a comprehensive set of parameters using three different swallowing positions.

This study aimed to investigate esophageal motility against gravity at the bridge position, using HRM. We also hypothesized that while in the position against gravity, the esophageal contractility of the esophagus would increase. Different from the Trendelenburg position, this study investigated swallowing in the bridge position using a cushion to create inversion, rather than using a specialized equipment. Specifically, we assumed that the bridge position might enhance esophageal motility more than the supine position.

## Materials and Methods

### Participants

Thirteen healthy subjects participated in the study. None of the subjects had a history of dysphagia, gastrointestinal disease, or other significant medical conditions. In addition, none of the subjects showed GERD symptoms or took antacids, including proton pump inhibitors or histamine H2 receptor antagonists. The study was approved by the Ethical Committee of Hamamatsu City Rehabilitation Hospital (Permission number: 18-47). Informed consent was obtained from all subjects before enrollment in the study.

### Study Design

A solid-state, high-resolution manometer (Starlet High-Resolution Manometry System, Star Medical, Tokyo, Japan) was used for the measurement. For this, a catheter (Unisensor AG, Attikon, Switzerland) with an outer diameter of 4.2 mm and 36 circumferential pressure sensors were spaced 1 cm apart. The sensors were unidirectional and were covered by circumferential soft membranes with fluid inside.

**Fig. 1:**
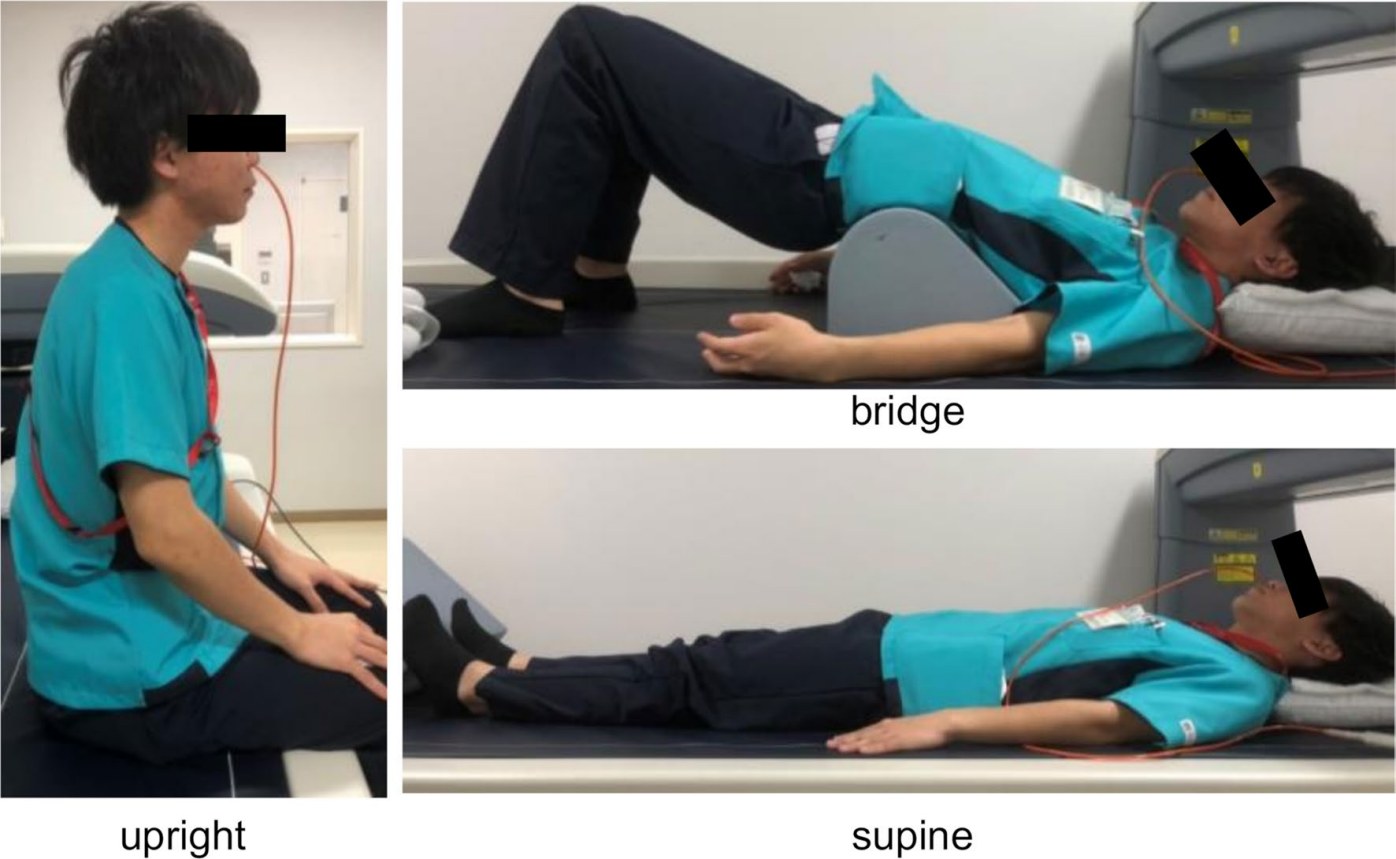
The body positions illustrated: upright; supine; bridge

Each participant swallowed 5 mL of cold water in the upright, supine, and bridge positions (Fig. 1). To change from a supine position to a bridge position, the participants bent their knees, raised their waist, and a cushion was then placed under their lower back (lower back support position). Studies were performed after at least 4 h of fasting. Before starting the examination, 2% lidocaine jelly was applied to the nasal passages. The catheter was also lubricated with lidocaine jelly to ease its passage through the nasal cavity and to reduce the discomfort of catheter insertion as much as possible. The catheter was calibrated and zeroed to atmospheric pressure. It was inserted trans-nasally until at least a few sensors were in the stomach and secured by taping it to the nose to record the pressure from the hypopharynx to the stomach. Before the assessment, the subjects rested for 5 min to acclimate to the presence of the catheter. Each subject then swallowed a 5 mL bolus of water, delivered orally via a syringe. Swallowing was repeated 5 times at 30-s intervals in the upright, supine, and bridge positions. Each subject conducted the swallowing tests in the same sequence of positions in order of upright, supine, and bridge.

### Outcome Variables

**Fig. 2:**
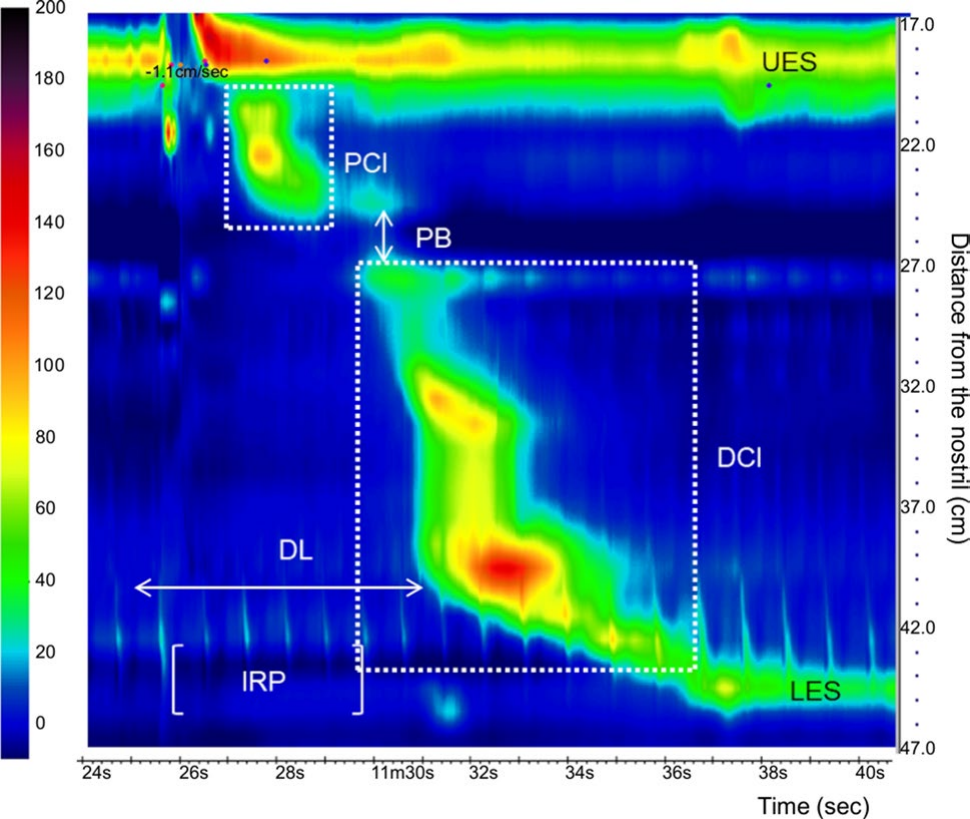
Pressure topography with time on the x-axis and distance from the nostril on the Y-axis. Pressure is indicated by the color scale. Resting upper esophageal sphincter (UES) and lower esophageal sphincter (LES) pressures are seen as horizontal bands of color that are several centimeters wide. The proximal contractile integral (PCI), distal contractile integral (DCI), integrated relaxation pressure (IRP), distal latency (DL) and peristaltic breaks (PBs) were measured

The manometric data were analyzed using HR-stealth software (Star Medical, Tokyo, Japan). Swallowing with more than one deglutition or accompanied with cough were rejected. The results are shown as pressure topography (Fig. 2).

The proximal contractile integral (PCI) value was calculated as the product of the mean amplitude of contractions in the proximal esophagus (mmHg) and the duration of contractions (s) for the length of the proximal esophageal segment (cm) exceeding 30 mmHg for the region spanning under the upper esophageal sphincter to the transition zone. The distal contractile integral (DCI) value was calculated as the product of the mean amplitude of contractions in the distal esophagus (mmHg) and the duration of contractions (s) for the length of the distal esophageal segment (cm) exceeding 30 mmHg for the region spanning from the transition zone to the proximal aspect of the LES. The integrated relaxation pressure (IRP) measured the mean pressure during 4 s of the greatest post-deglutitive relaxation in a 10-s gap, triggered at the beginning of a swallow, which corresponds to the relaxation of the LES. Distal latency (DL) was measured as the peristalsis velocity from the beginning of a swallow to the epiphrenic ampulla. Peristaltic breaks (PBs) were assessed by measuring the length of axial breaks in the 30 mmHg isobaric contour between the proximal esophagus and distal esophagus. The inspiratory EGJ pressure was measured as the mean of maximal EGJ pressures during three respiratory cycles. The expiratory EGJ pressure was measured by the average EGJ pressure midway between adjacent inspirations for three respiratory cycles. The EGJ pressure and gastric pressure (GP) were measured three times for each position. Intrabolus pressure (IBP) was measured between the peristaltic wave front and the EGJ. GP was measured 2 cm below the EGJ.

### Statistical Analysis

The PCI and DCI values, IRP, DL, and PBs were measured, and comparisons between the positions were analyzed. The Chicago Classification 3.0 criteria were used to characterize esophageal motility using pressure topography parameters [19]. Comparisons were performed using the Friedman analysis and the Steel–Dwass method as the post hoc test. The critical value for rejecting the null hypothesis was *p* < 0.05. All statistical analyses were performed using the IBM SPSS statistics 25.0 software (IBM Japan Corp., Tokyo, Japan).

## Results

### Participant Characteristics

In total, 8 males and 5 females (mean age 34 years, range 23–64) participated in the study. According to the Chicago Classification diagnoses for the 13 subjects (based on the five supine swallows), 11 participants were categorized as normal and 2 were diagnosed with ineffective esophageal motility (IEM) [19].

**Fig. 3:**
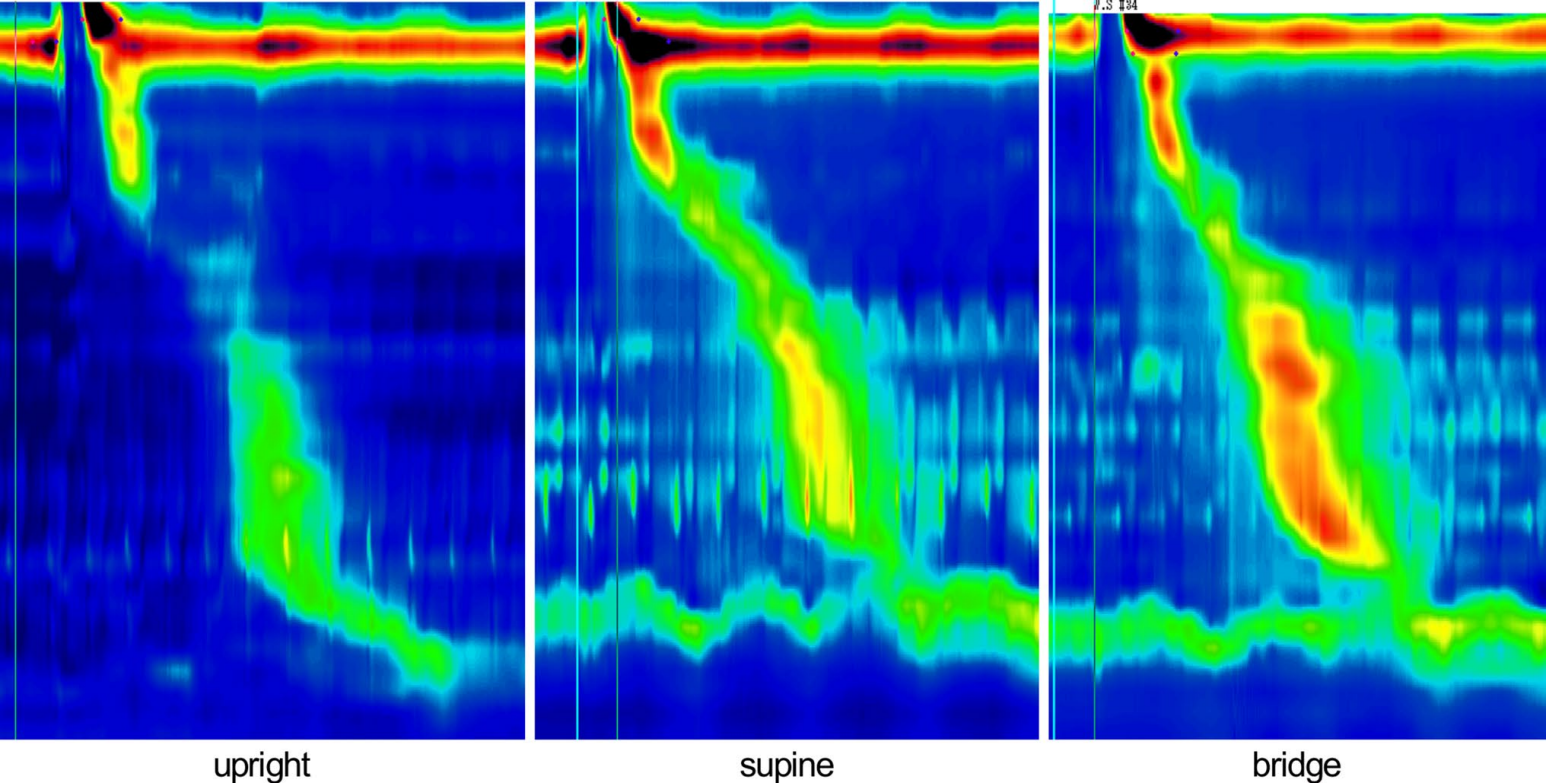
Pressure topography in three different body positions (upright; supine; bridge) while one subject swallowed 5 mL of water

Figure 3 shows the pressure patterns in each of the 3 positions for one participant (21-year-old male). A summary of the descriptive statistics, with the results of the main effects, is provided in Table 1.

**Table 1:** Summary data of esophageal motility in the upright, supine, and bridge position (mean ± SD)

Parameter	Upright	Supine	Bridge	*p* value	Post hoc test *p* value		
					Upright/supine	Upright/bridge	Supine/bridge
PCI (mmHg-cm-s)	306.4 ± 144.6	694.0 ± 317.4	683.1 ± 365.7	< 0.001*	< 0.001*	0.001*	1.000
DCI (mmHg-cm-s)	971.5 ± 889.1	1783.3 ± 1404.3	2763.2 ± 1392.2	< 0.001*	0.233	< 0.001*	0.056
DL (s)	6.3 ± 2.1	6.9 ± 2.2	8.2 ± 1.0	0.001*	0.350	0.001*	0.093
PB (cm)	6.3 ± 5.3	3.1 ± 4.9	0.3 ± 0.6	< 0.001*	0.018*	0.001*	0.980
IRP (mmHg)	8.6 ± 7.5	13.3 ± 8.5	16.4 ± 9.3	0.018*	0.150	0.018*	1.000
Expiratory EGJ pressure (mmHg)	11.0 ± 6.2	18.2 ± 5.5	28.3 ± 8.1	0.001*	0.350	0.001*	0.093
Inspiratory EGJ pressure (mmHg)	38.0 ± 10.3	47.2 ± 14.1	63.0 ± 18.5	< 0.001*	0.718	< 0.001*	0.010*
Expiratory GP (mmHg)	5.6 ± 4.7	5.2 ± 3.2	10.5 ± 5.3	0.006*	0.980	0.093	0.005*
Inspiratory GP (mmHg)	9.4 ± 5.4	8.3 ± 2.8	15.4 ± 7.0	0.023*	1.000	0.093	0.032*
IBP (mmHg)	8.8 ± 8.5	7.1 ± 6.0	14.5 ± 7.8	0.008*	1.000	0.056	0.010*

*PCI* proximal contractile integral, *DCI* distal contractile integral, *DL* distal latency, *PB* peristaltic breaks, *IRP* integrated relaxation pressure, *EGJ* esophagoesophageal junction, *GP* gastric pressure, *IBP* intrabolus pressure

^*^Significant *p* values by Friedman analyses and Post hoc test

### Outcomes

The PCI was significantly higher in the supine and bridge positions than in the upright position (*p* < 0.001 and *p* = 0.001, respectively). There were no significant differences in the PCI values between the supine and bridge positions. The DCI value was significantly higher in the bridge than in the upright position (*p* < 0.001). The DCI value in the bridge position was numerically but insignificantly higher than in the supine (*p* > 0.05). There were no significant differences in the DCI value between the upright and supine positions. The IRP was significantly higher in the bridge than in the upright position (*p* = 0.018). There were no significant differences between the upright and supine positions, and no significant differences were noted between the bridge and supine positions in IRP. The expiratory and inspiratory EGJ pressures were significantly higher in the bridge than in the upright position (expiratory: *p* = 0.001) (inspiratory: *p* < 0.001). The IBP was significantly higher in the bridge than in the supine position (*p* = 0.01). DL was significantly longer in the bridge than in the upright position (*p* = 0.001). There were no significant differences between the upright or bridge and supine positions. The PBs were significantly shorter in the bridge than in the upright position (*p* = 0.001). PBs were also significantly shorter in the supine than in the upright position (*p* = 0.018). There were no significant differences in PBs between the supine and bridge positions.

## Discussion

This study aimed to measure esophageal motility, including that of the distal esophagus and EGJ, during swallowing while in an upright, supine, and bridge position using HRM. The most important finding of the study is that esophageal peristalsis was significantly stronger in the distal esophagus while in the bridge position, which was against gravity.

Rosen et al. [16] found that there were no significant differences in pressure integral values in the proximal esophagus in the supine and inverted positions using an inversion table. Although the experimental conditions were different from those of the present study, the similar results with those of our study confirm that the proximal esophagus may be influenced by other factors, regardless of the body position. On the other hand, Zifan et al. [18] observed that contractile integrals in the proximal and distal esophagus were also significantly higher in the Trendelenburg position than in the supine, to transport the bolus against gravity. The question remains whether proximal esophageal peristalsis is stronger when against gravity.

When comparing the results from the three body positions, the DCI values tended to increase in the following order: upright < supine < bridge. Similar to previous studies [7–10], the DCI tended to be higher in the supine position compared to the sitting position, but there was no statistically significant difference. This might be due to differences in sample size or statistical analysis methods between this study and previous studies. The results also indicated that esophageal peristalsis was significantly stronger when going against gravity. In our study, the DCI value in the supine position was lower than that reported in a previous study using the same catheter [20]. This might be due to the influence of the 2 subjects diagnosed with IEM within the small sample size. Although IEM can be found in healthy subjects, further study is needed to elucidate the effect of gravity on esophageal motility in these subjects [19].

In this study, PBs were significantly shorter in the bridge position, which may be due to the strong contractions of the esophagus when working against gravity. Similar to our study, Zifan et al. [18] identified that the breaks in peristalsis were reduced in the Trendelenburg position. They reported that due to the increased distension in the proximal esophagus while in the Trendelenburg position, the length of breaks was markedly reduced and often disappeared. In the present study, shorter PB might also reflect enhanced contractility of the proximal esophagus, affected by posture.

The second important finding is that the IRP in the bridge position was significantly higher than in the upright position. Similar to the DCI value, the results demonstrate that the IRP was higher when going against the force of gravity. In addition to IRP, expiratory and inspiratory EGJ pressures (free of swallows) were also significantly higher in the bridge position than those in the upright, and they tended to increase in the following order: upright < supine < bridge. This result is similar to that of a previous study, in which the Trendelenburg position increased the LES pressure as a protective mechanism to prevent the reflux of gastric contents [17].

Of note, the significantly higher gastric pressure in the bridge position, compared to the supine gastric pressure, probably indicates increased diaphragmatic and/or abdominal wall muscle tone in the bridge position (it is not as passive as the supine or Trendelenburg positions). Comparing the bridge position with the supine, increased gastric pressure resulted in a significantly increased EGJ pressure and IBP, but IRP (gradient between IBP and gastric pressure) remained stable.

Another point of note was that the DL in the bridge position was significantly longer than that in the upright position; this may be because esophageal peristalsis occurred slower when against the gravity.

In the esophageal phase, the primary peristaltic waves occur after swallowing. This is an important mechanism for acid clearance from the esophagus, which when not properly functioning, causes excessive esophageal acid exposure [21].

Our findings indicate that esophageal contractions become stronger against the force of gravity, and the bridge posture can be easily reproduced. Currently, exercises to facilitate esophageal motility for dysphagia patients are not widely known, but there is potential for using this technique to facilitate esophageal stage swallowing. This may be of clinical importance for improving patient outcomes. However, the physiology related to increased esophageal peristalsis is poorly understood, and if swallowing training in the bridge position is performed it is unknown how long the effect will continue. Therefore, more studies are required to examine the long-term effect of swallowing in the bridge position.

The present study has several limitations to consider. First, data were collected in a relatively small number of participants and included relatively young and healthy subjects; thus, the results are not generalizable. Further studies should include a larger sample size and participants with GERD, in addition to healthy controls. The second limitation was that usually 10 swallows are obtained in clinical esophageal manometry for an accurate clinical diagnosis, but only 5 swallows were performed in this study. This was to minimize the burden on the subjects since all the subjects performed five swallows in each position for the study. The third was that this study did not measure the body angle in each subject while in the different positions. Finally, the present study focused on manometric data without examining the bolus passage, which can be detected by videofluoroscopic studies.

## Conclusions

Our findings indicate that distal esophageal contractions are stronger and progress slower against the gravitational force.
